# Impact of the COVID‐19 pandemic on HIV prevention and care services among key populations across 15 cities in India: a longitudinal assessment of clinic‐based data

**DOI:** 10.1002/jia2.25960

**Published:** 2022-07-11

**Authors:** Allison M. McFall, Neia Prata Menezes, Aylur K. Srikrishnan, Sunil S. Solomon, Santhanam Anand, Jiban J. Baishya, Gregory M. Lucas, David D. Celentano, Shruti H. Mehta

**Affiliations:** ^1^ Department of Epidemiology Johns Hopkins Bloomberg School of Public Health Baltimore Maryland USA; ^2^ YR Gaitonde Centre for AIDS Research and Education Chennai India; ^3^ Department of Infectious Diseases Johns Hopkins University School of Medicine Baltimore Maryland USA

**Keywords:** India, COVID‐19, health services needs and demand, HIV testing, men who have sex with men, people who inject drugs

## Abstract

**Introduction:**

The COVID‐19 pandemic has threatened to diminish gains in HIV epidemic control and impacts are likely most profound among key populations in resource‐limited settings. We aimed to understand the pandemic's impact on HIV‐related service utilization among men who have sex with men (MSM) and people who inject drugs (PWID) across India.

**Methods:**

Beginning in 2013, we established integrated care centres (ICCs) which provide HIV preventive and treatment services to MSM and PWID across 15 Indian sites. We examined utilization patterns for an 18‐month period covering 2 months preceding the pandemic (January–February 2020) and over the first and second COVID‐19 waves in India (March 2020–June 2021). We assessed: (1) unique clients accessing any ICC service, (2) ICC services provided, (3) unique clients tested for HIV and (4) HIV diagnoses and test positivity. Among an established cohort of PWID/MSM living with HIV (PLHIV), we administered a survey on the pandemic's impact on HIV care and treatment (June–August 2020).

**Results:**

Overall, 13,854 unique clients visited an ICC from January 2020 to June 2021. In January/February 2020, the average monthly number of clients was 3761. Compared to pre‐pandemic levels, the number of clients receiving services declined sharply in March 2020, dropping to 25% of pre‐pandemic levels in April/May 2020 (first wave), followed by a slow rebound until April/May 2021 (second wave), when there was a 57% decline. HIV testing followed a similar trajectory. HIV test positivity changed over time, declining in the first wave and reaching its nadir around July 2020 at ∼50% of pre‐pandemic levels. Positivity then increased steadily, eventually becoming higher than pre‐pandemic periods. The second wave was associated with a decline in positivity for MSM but was relatively unchanged for PWID. Among 1650 PLHIV surveyed, 52% of PWID and 45% of MSM reported the pandemic impacted their ability to see an HIV provider. MSM had barriers accessing sexually transmitted infection testing and partner HIV testing.

**Conclusions:**

The COVID‐19 pandemic led to significant decreases in HIV‐related service utilization among key populations in India. This presents an opportunity for increased transmission and patients presenting with advanced disease among groups already disproportionately impacted by HIV.

## 1 INTRODUCTION

As of 18 May 2022, there have been more than 520 million confirmed COVID‐19 cases (the disease caused by the SARS‐CoV‐2 virus) globally [1]. The United States has reported the highest number of cases followed by India with over 40 million cases [1]. There are concerns about the impact the COVID‐19 pandemic and its associated lockdowns have had on access to preventive and treatment services for other infectious diseases and the potential negative impact on the 2030 Sustainable Development Goals and elimination targets, including the 2030 UNAIDS fast‐track targets. Key populations, such as people who inject drugs (PWID) and men who have sex with men (MSM), are particularly vulnerable given underlying individual and structural barriers to engaging in HIV treatment and preventive services. For example, these populations in India face economic and social barriers [2, 3] that could impact access to telemedicine and lead to a reliance on public transportation for reaching health centres, and tend to have a high burden of mental health comorbidity and substance use [4, 5], which may have been exacerbated by the pandemic.

In India, the first cases of SARS‐CoV‐2 were reported in late‐January 2020 [6]. India implemented a strict nationwide lockdown early on from March to May 2020 [7, 8]. The lockdown impacted public transportation and travel; specifically, no public transportation was operational and travel was only allowed for essential services during restricted hours. Impacts on HIV services resulted from the repurposing of many government facilities/staff that provide HIV services to COVID‐19 services. After May 2020, lockdowns were variably implemented across and within states. With the second more devasting COVID wave beginning in March 2021, and peaking in early May 2021 with more than 400,000 documented cases per day, came renewed partial lockdowns and restrictions on movement in most states. These were regional lockdowns, dictated by local case rates, and generally not as restrictive as those imposed in the initial wave. With a few exceptions (e.g. northeastern India), most restrictions were lifted by mid‐June 2021.

Early in the pandemic, India's National AIDS Control Program rapidly re‐designed components of their program to ensure service continuity [9]. For example, in India, public‐sector antiretroviral therapy (ART) has traditionally been dispensed in 30‐day intervals through government facilities. However, in response to restrictions, the program pivoted to multi‐month dispensing (MMD)—typically 2–3 months’ worth—for persons living with HIV (PLHIV). In some settings, there was further provision for home/community‐based delivery of ART. Additionally, medication for opioid use disorder (MOUD) moved from requiring daily dispensation towards 5–7 day dispensations.

Little is known regarding the impact of COVID‐19 on other HIV‐related service provision outside government facilities especially among key populations [10]. We examined pandemic impact by assessing utilization patterns at MSM and PWID‐focused, community‐based care centres from the 2 months preceding the pandemic (January–February 2020) through the second COVID‐19 wave in India (March 2020–June 2021).

## 2 METHODS

We leveraged data from two related sources. First, we examined service utilization data from 16 integrated care centres (ICCs) in 15 cities that provided HIV‐related services to either MSM (cities: Delhi, Bangalore, Belgaum, Hyderabad, Bhopal, Vijayawada, Visakhapatnam and Madurai) or PWID (cities: Delhi, Ludhiana, Amritsar, Bilaspur, Kanpur, Churachandpur, Aizawl and Dimapur). Second, we conducted telephone/web‐based surveys among PLHIV ICC clients who were part of a cluster randomized trial.

### 2.1 ICC service utilization data

We established ICCs between 2013 and 2018 as public–private partnerships representing a collaboration between Johns Hopkins University, an Indian non‐governmental organization YR Gaitonde Centre for AIDS Research and Education (YRGCARE) and the National AIDS Control Organization, India as previously described [11, 12]. ICCs are open 6–7 days a week and staffed by a supervisor, nurse, counsellor, phlebotomist, part‐time physician and outreach workers. ICCs were specifically designed to provide comprehensive population‐focused HIV services for PWID or MSM. ICCs offer point‐of‐care rapid HIV testing according to the national guidelines, counselling (e.g. risk reduction [safe sex/injection], substance use, depression, family/couple counselling and ART adherence), tuberculosis screening and general medical examinations, and key‐population‐focused services (testing and syndromic treatment of sexually transmitted infections [STIs], hepatitis C virus testing, condom distribution, MOUD and field‐based needle/syringe exchange). ICC staff provide support for ART initiation and refills, including accompanying clients to government ART centres, which are often located in contiguous space. Only one ICC (Aizawl) provides ART refills; none initiate ART. ICC commodities are provided by the National/State AIDS Control Programs and staffing is supported by the research program. All services are free to clients.

Most ICCs experienced some change in service availability during the COVID‐19 pandemic but this was highly variable and related to local epidemic severity. For example, in Delhi where the pandemic was most severe, MSM and PWID ICCs were shutdown from mid‐March to June 2020. By contrast, in Kanpur, other services were stopped, while MOUD and ART refill linkages were provided. In Ludhiana and Amritsar, ICCs remained fully functional over the entire period. The Madurai MSM ICC closed for in‐person services mid‐March–May 2020 but provided counselling support and linkage to services via mobile phone and ART pill pick‐up using outreach workers. Other MSM ICCs remained open throughout this period with variable interruptions in service coverage.

ICCs used a fingerprint‐based biometric system for identifying unique clients and tracking service utilization. ICC staff record service data and test results in an encrypted, cloud‐based centralized database. For client and staff safety with respect to SARS‐CoV‐2 transmission, and as directed by the government of India, the fingerprint‐based biometric system was suspended beginning mid‐March 2020. Tracking of most ICC services, however, continued and client identity was confirmed using identifiable information, such as name, age and mobile number or address. Biometric systems utilizing a non‐contact iris system were resumed from October 2021.

### 2.2 COVID‐19 impact surveys with PLHIV

At the onset of the COVID‐19 pandemic, we were in the midst of conducting a cluster‐randomized trial at the 16 ICCs to evaluate the effectiveness of incentives for HIV care and treatment (e.g. picking up ART refills at the government centre) to increase viral suppression (www.ClinicalTrials.gov identifier: NCT02969915) [11, 13]; eight ICCs were randomized to provide incentives. Between October 2017 and October 2018, we recruited approximately 150 participants at each ICC, with follow‐up visits planned every 6 months for 24 months. Inclusion criteria included being 18 years or older, HIV positive and either ART‐naïve or prescribed ART for <12 months. Research activities for the trial (i.e. in‐person study visits and incentives) were stopped 16 March 2020. Between June and August 2020, study staff reached out to cohort participants for whom we had telephone numbers and consent to contact, to request participation in a survey. Participants completed the survey over the telephone with research staff, or via a web link, and were reimbursed for their time. The survey took approximately 25 minutes and included questions on COVID‐19 symptoms/testing, pandemic effects on healthcare visits, ART access, adherence to HIV treatment and access to preventive services, including MOUD, clean needles/syringes and STI testing.

### 2.3 Ethics

This study was approved by institutional review boards at Johns Hopkins University School of Medicine and YRGCARE. A waiver of consent was granted for analyses of ICC client service utilization data. Cohort participants provided written informed consent.

### 2.4 Statistical analysis

To assess the impact of the COVID‐19 pandemic on service utilization, we examined ICC utilization patterns for an 18‐month period covering the 2 months preceding the pandemic (January–February 2020) and then over the first and second COVID‐19 waves in India (March 2020–June 2021). Prior to the pandemic, ICC utilization was relatively stable over time with no substantial seasonal variation making January/February 2020 representative of pre‐pandemic utilization. We assessed the numbers of: (1) unique clients accessing any ICC service, (2) different ICC services provided, (3) unique clients tested for HIV and (4) HIV positivity (number of HIV diagnoses/number of HIV tests). The assessment of different ICC services provided was a count of services—not unique clients; clients could be represented multiple times, if they used a service more than once in a month. MOUD was excluded due to challenges collecting accurate utilization data on the higher volume of MOUD clients without the use of the biometric system earlier in the pandemic, and changing guidelines around dispensation of MOUD. ART refills were excluded from all analyses since only one ICC directly dispensed ART and ART data abstraction from government books was inconsistent over the analysis period.

Service data were aggregated monthly and then summarized by population group (PWID/MSM); any service use among unique clients was further summarized by site. To quantify and visualize the pandemic impact on ICC service utilization, we calculated and graphed the percent difference in number of unique clients compared to January/February 2020 and used a 2‐month moving average to smooth month‐to‐month fluctuations. HIV positivity was also graphed using a 2‐month moving average. Additionally, we used negative binomial regression models to statistically compare temporal trends of any ICC service use and HIV testing by COVID‐19 pandemic periods; detailed methods are in Supplementary Material.

For PLHIV who completed the COVID impact survey, experiences with COVID‐19 symptoms/testing and their use of HIV related, non‐HIV related and prevention/harm reduction services during the pandemic were summarized using descriptive statistics.

## 3 RESULTS

Overall, 13,854 unique clients visited an ICC from January 2020 to June 2021 (6964 MSM and 6886 PWID). Median age was 30 years (MSM 30 and PWID 31), 94% of PWID were men, and at registration 38% were married (MSM 33% and PWID 44%), 26% unemployed (MSM 25% and PWID 27%) and 31% were daily wage earners (MSM 27% and PWID 35%). Overall, 4684 (34%) acquired HIV as of June 2021 (MSM 28% and PWID 39%).

### 3.1 Temporal trends in service utilization

**Figure 1:**
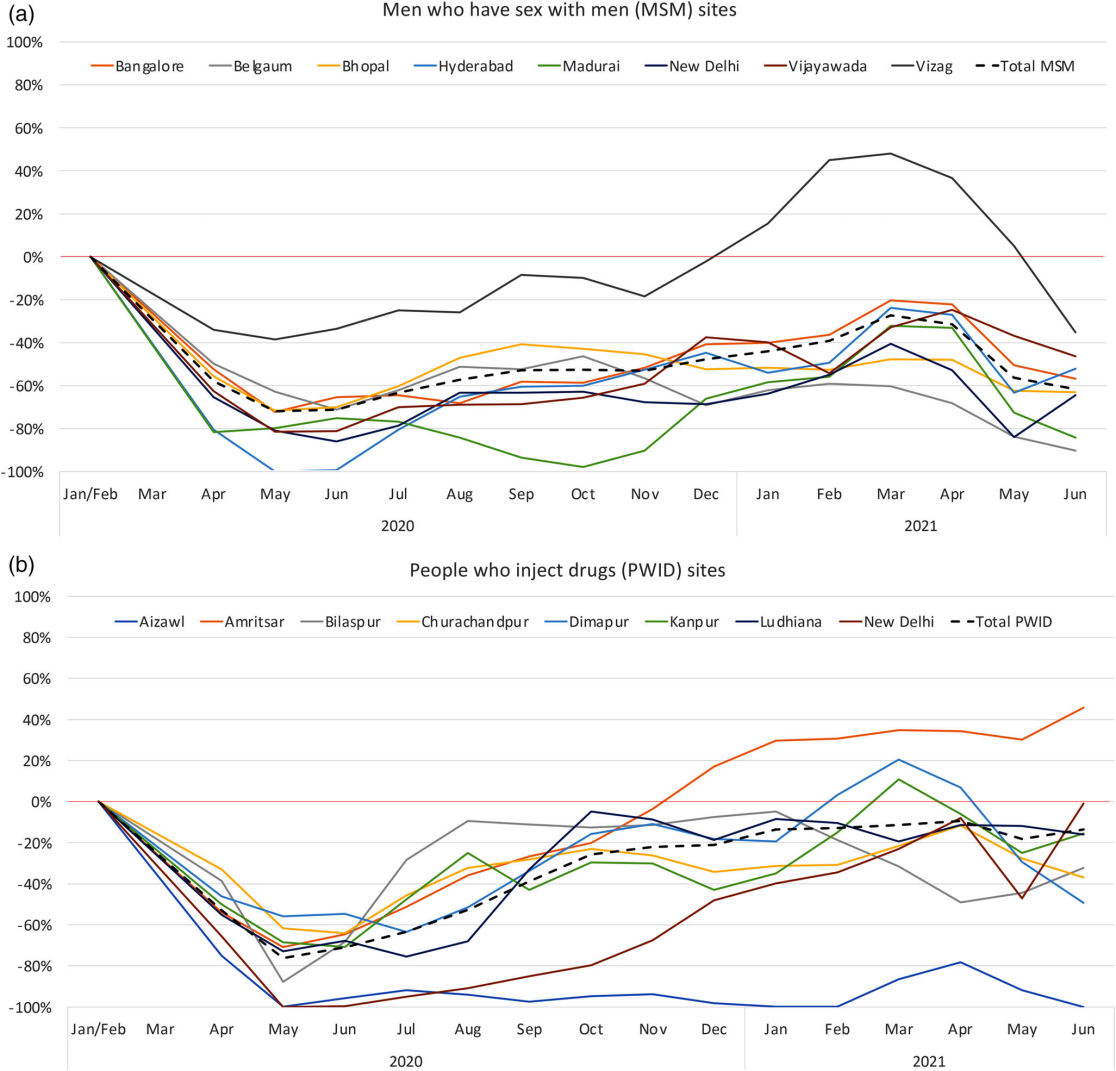
Percentage difference in integrated care centre service utilization (for any reason) among unique clients relative to the pre‐COVID‐19 pandemic period (January–February 2020) using a 2‐month moving average, by site/city, among men who have sex with men (Panel a) and people who inject drugs (Panel b).

The average monthly number of unique ICC clients in January/February 2020 (pre‐pandemic) was 3761 (*n* = 1456 MSM and *n* = 2305 PWID). Compared to pre‐pandemic levels, the overall number of clients receiving any type of service at the ICCs began a sharp decline in mid‐March 2020 (beginning of first wave) and dropped to 25% of pre‐pandemic levels in April/May (Figure S1). There was a slow but steady rebound to 87% of pre‐pandemic levels in March 2021 until April/May 2021, when the second COVID‐19 wave began. Utilization dropped to 57% of pre‐pandemic levels in May 2021 followed by a rebound in June 2021. Figure 1 shows the percentage difference compared to January/February 2020 in any service utilization. The initial decline due to the first wave was similar for PWID and MSM. Following this initial decline, however, rebound was slower for MSM (Figure 1a) versus PWID ICCs (Figure 1b) so that by March 2021, MSM utilization was at 77% of pre‐pandemic levels, while PWID utilization was almost back to pre‐pandemic levels (94%). The second pandemic wave in May 2021 was associated with a larger drop in utilization among MSM than PWID (28% vs. 76% of pre‐pandemic levels). Declines in both waves were statistically significant for MSM and PWID (Table S1). Utilization patterns by city were fairly consistent with the notable exception that the PWID ICCs in Ludhiana and Amritsar did not have substantial declines associated with the second wave.

**Figure 2:**
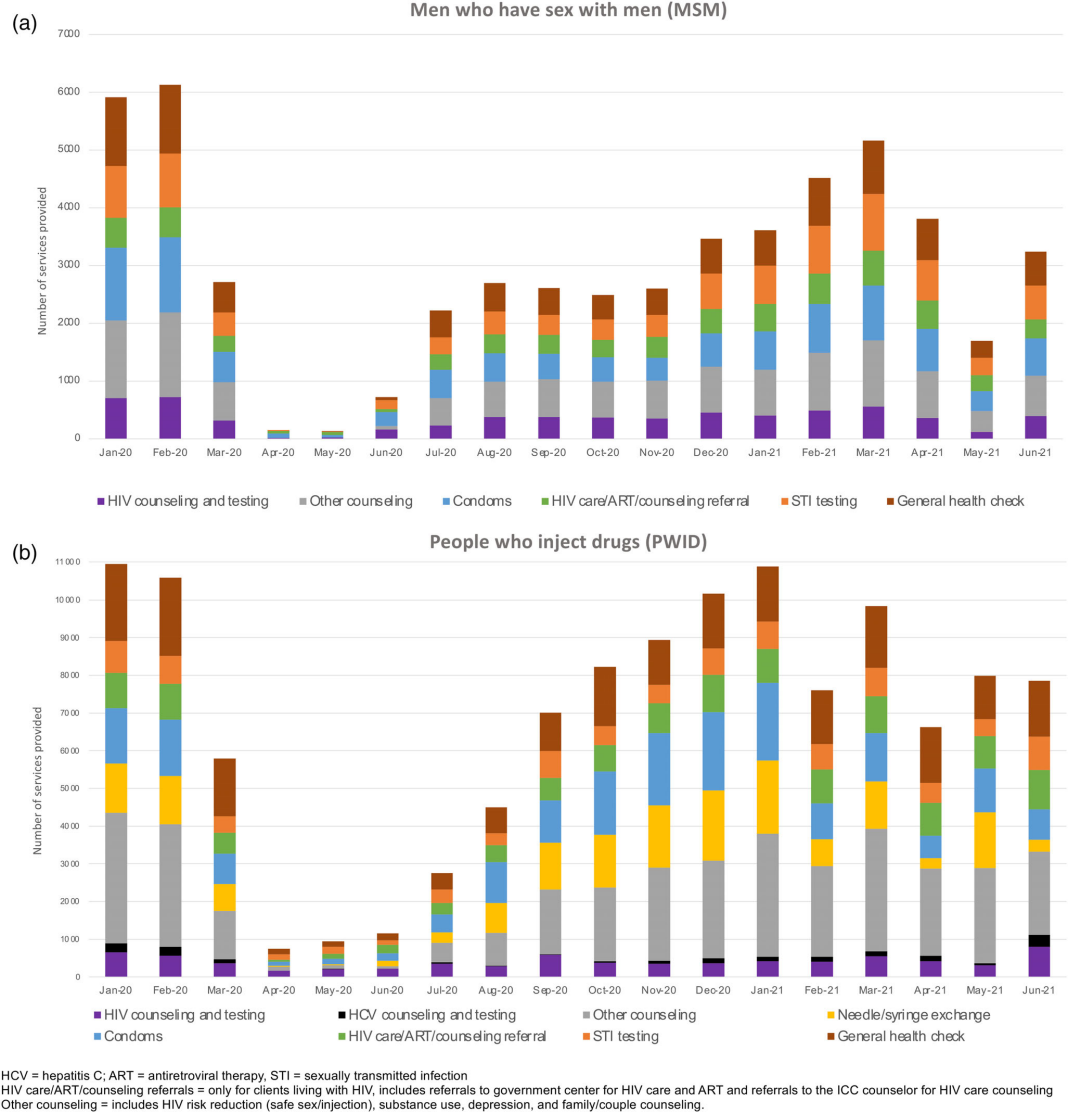
Services utilized at integrated care centres from January 2020 to June 2021 among men who have sex with men (Panel a) and people who inject drugs (Panel b). Persons could utilize more than one service at a given point in time.

In the pre‐pandemic period, on average, 16,790 services were provided each month (*n* = 6024 for MSM and *n* = 10,766 for PWID), and the primary ICC services utilized by both MSM and PWID were counselling and general health check‐ups (Figure 2a,b). Declines were experienced uniformly across services with commensurate rebounds to pre‐pandemic levels by January–March 2021. In the second wave, the only service that appeared to be disproportionately affected relative to other services was needle/syringe exchange. There was a decline of ∼90% in HIV care/treatment referrals for both MSM and PWID during the first wave of the pandemic with a rebound by December 2020 (PWID) and February 2021 (MSM). Declines in referrals during the second wave were more severe for MSM (almost 50% in May 2021 compared to pre‐pandemic) than for PWID (9%).

**Figure 3:**
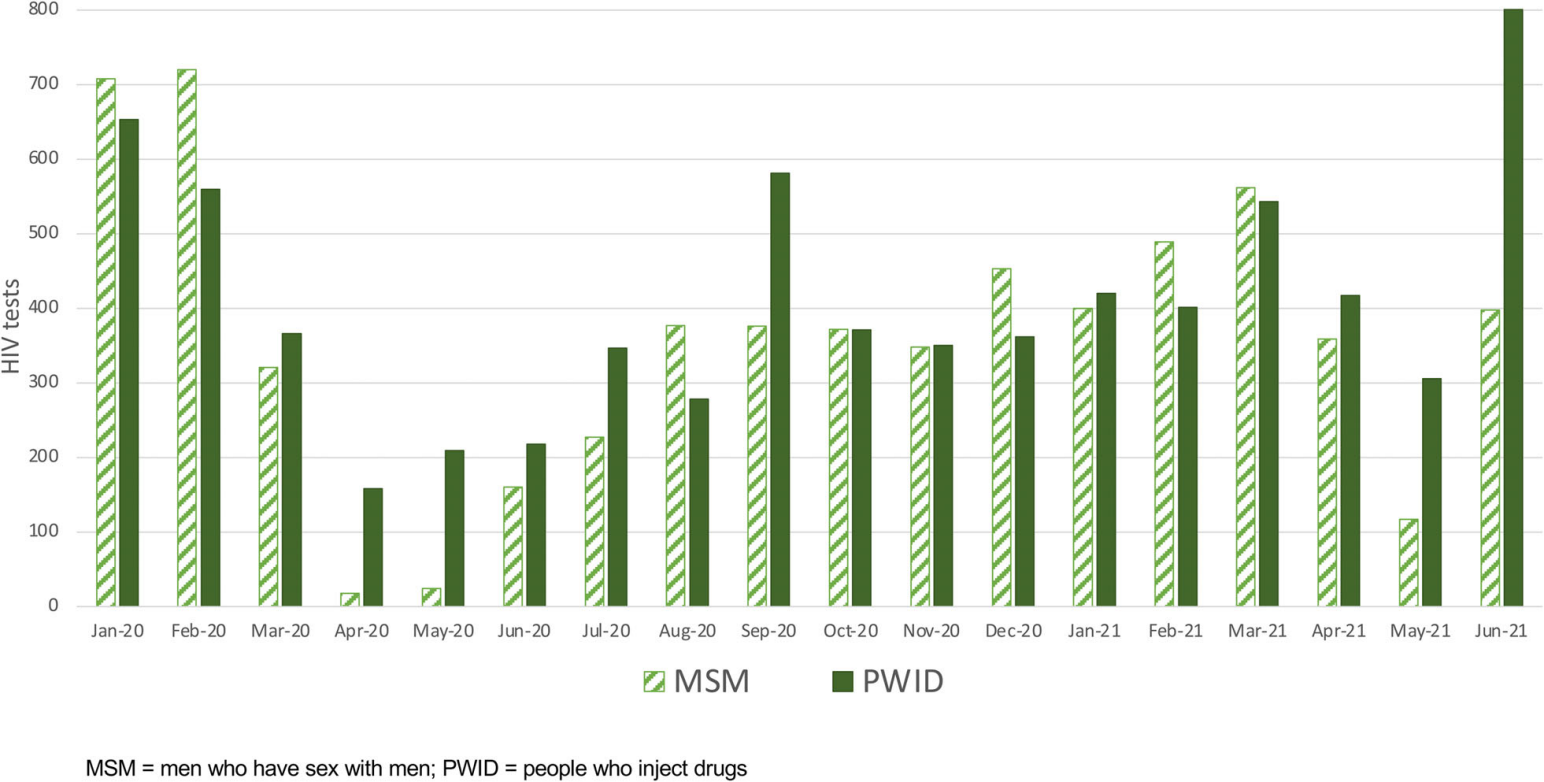
Number of unique clients receiving HIV tests from January 2020 to June 2021 among men who have sex with men and people who inject drugs.

The average monthly number of unique clients who received HIV testing in January/February 2020 was 1321 (*n* = 714 MSM and *n* = 607 PWID). The numbers of clients receiving HIV tests dropped to 13% of pre‐pandemic levels in April 2020 (3% and 26% for MSM and PWID, respectively) (Figure 3). HIV testing gradually increased over the next 11 months though never fully returning to pre‐pandemic levels by March 2021 (84% of pre‐pandemic levels overall, 79% and 90% for MSM and PWID, respectively). Testing dropped again in May 2021 during the second COVID‐19 wave—32% of pre‐pandemic levels overall; 16% and 51% for MSM and PWID, respectively. The declines during these two waves were statistically significant for MSM and PWID (Table S2).

**Figure 4:**
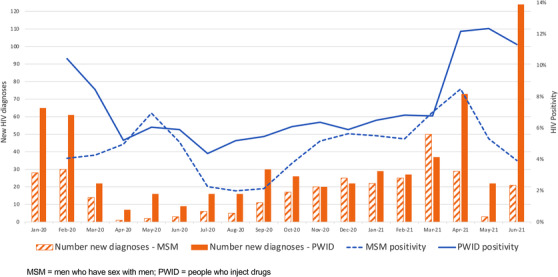
Number of HIV diagnoses and test positivity (2‐month moving average) from January 2020 to June 2021 among men who have sex with men and people who inject drugs.

For January/February 2020, the average monthly HIV test positivity was 7.0% (4.1% for MSM and 10.4% for PWID). For PWID, there was a sharp decline in positivity during the first wave. For MSM, there was a slight increase; however, there were relatively fewer tests—42 tests total in April/May 2020 versus ∼700/month in February/January 2020 (Figure 4). The decline in diagnoses was especially severe in the 5–6 months following the start of the pandemic. Positivity reached its nadir around July 2020 at approximately half of the pre‐pandemic period and then increased steadily, eventually becoming higher than pre‐pandemic periods by October/November 2020 for MSM and by March 2021 for PWID. Positivity again dropped during the second wave for MSM from 9% in April 2021 down to 5% in May 2021; it was relatively unchanged for PWID (12% in April and May 2021).

### 3.2 Barriers to accessing HIV preventive and treatment services during the first COVID‐19 wave

Among 2314 PLHIV enrolled in the parent cluster‐randomized trial, 234 (10%) had died as of 15 March 2020, leaving 2080 eligible for the COVID‐impact survey. Of 1054 from PWID sites, 758 (72%) completed a survey, of 1026 from MSM sites, 892 (87%) completed a survey. Compared to those who did not complete the survey, participants who completed it were more likely to have been on ART at their prior cohort visit (55% vs. 90%).

Among 1650 PLHIV surveyed, median age was 32, 46% were currently married, 12% of the PWID were women and 19% were unemployed prior to the COVID‐19 pandemic. At the time of the survey, 81 (9%) of MSM reported ever having symptoms of COVID‐19, of whom 19 (23%) reported being tested for SARS‐CoV‐2; 138 (18%) PWID reported ever having symptoms, of whom three (2%) reported being tested for SARS‐CoV‐2. No MSM or PWID reported testing positive for SARS‐CoV‐2.

When asked about how the pandemic impacted HIV care, 52% of PWID and 45% of MSM reported that the pandemic had impacted their ability to see an HIV provider (Table 1). PWID were more likely than MSM to report missing HIV care appointments (24% vs. 13%) and ART doses (28% vs. 17%). Among PWID, men and women missed HIV care appointments to the same extent (15%) but men were more likely to report missed ART doses than women (31% vs. 13%, respectively). For both MSM and PWID, the main reason for non‐adherence to ART was the inability to get to a treatment centre due to COVID‐19 shutdowns/closures and wanting to avoid public transportation, followed by forgetting to take the medication. For prevention services, 60% of MSM who needed an STI test could not get one and 47% of MSM who needed HIV testing for partners could not access it. By contrast, fewer PWID reported an inability to access key services like needle/syringe services (12%) and MOUD (8%).

**Table 1:** Self‐reported heath service engagement and associated barriers among MSM and PWID living with HIV (June–August, 2020)

*n* (%)/median (IQR)	MSM (*N* = 892)	PWID (*N* = 758)
**HIV care and antiretroviral therapy (ART)‐related service engagement**		
Missed appointment for HIV care in the past 30 days	116 (13.0)	179 (23.6)
Difficulty in seeing an HIV care provider during the pandemic	404 (45.3)	390 (51.5)
ART use prior to/at the start of COVID‐19 pandemic	788 (88.0)	529 (69.7)
ART adherence <100% in the past 30 days	135 (17.0)	149 (28.2)
Major reasons for ART non‐adherence:		
*COVID‐19 shutdowns*	46 (34.0)	64 (42.9)
*Avoiding public transportation*	31 (23.0)	18 (12.1)
*Avoiding crowds at ART centres*	8 (5.9)	9 (6.0)
*Forgot*	9 (6.7)	17 (11.4)
*Don't need treatment*	11 (8.1)	5 (3.4)
Median days of ART in possession	30 (15–60)	30 (10–45)
**Harm reduction/preventive service needs and unmet needs in prior 30 days**		
STI testing needed	306 (34.3)	377 (50.7)
*Could not access STI testing*	182 (59.5)	44 (11.7)
HIV testing for spouses/partners needed	383 (43.0)	392 (51.7
*Could not access spousal/partner HIV testing*	180 (46.9)	35 (8.9)
Condoms needed	443 (49.6)	349 (46.1)
*Could not access condoms*	117 (26.4)	27 (7.2)
Lubricant needed	390 (23.6)	154 (20.3)
*Could not access lubricant*	90 (23.1)	31 (20.1)
Needles/syringes needed	97 (10.9)	343 (45.3)
*Could not access needles/syringes*	92 (94.8)	40 (11.7)
Medication for opioid use (MOUD) disorder needed	35 (3.9)	338 (44.6)
*Could not access MOUD*	10 (28.6)	28 (8.3)
**Non‐HIV health conditions service engagement**		
Missed medications in the prior 30 days	4 (8.3)	2 (1.9)
Missed an appointment with a non‐HIV health provider in prior 30 days	60 (6.7)	112 (14.8)
Major reasons for missing an appointment with a health provider		
*COVID‐19 shutdowns*	13 (21.7)	31 (27.7)
*Avoiding public transportation*	12 (20.0)	6 (5.4)
*Avoiding being around people*	9 (15.0)	27 (24.1)
*Felt okay*	14 (23.3)	26 (23.2)
Difficulty in seeing a non‐HIV care provider during the pandemic	331 (47.1)	371 (48.9)

Abbreviations: COVID‐19, disease caused by the SARS‐CoV‐2 virus; IQR, interquartile range (25th and 75th percentiles); MSM, men who have sex with men; PWID, people who inject drugs; STI, sexually transmitted infection.

## 4 DISCUSSION

Among key populations across multiple Indian states, we observed that nearly all HIV‐related services were dramatically affected by the COVID‐19 pandemic and associated lockdowns, particularly during the first wave of the pandemic. Impacts on HIV testing were large and generally more severe during the first versus the second wave—potentially due to less restrictive lockdowns and lessons learned resulting in better access. MSM also reported barriers to accessing testing for STIs and partner HIV testing. While HIV testing levels never fully rebounded to pre‐pandemic levels for MSM, they did for PWID and new diagnoses increased substantially during the second wave of the COVID‐19 pandemic. Collectively, these data suggest that the COVID‐19 pandemic and associated lockdowns may have led to delayed and missed HIV diagnoses, which may contribute to increased HIV transmission and more people presenting with advanced disease among vulnerable groups.

Our data are consistent with other reports demonstrating the negative impact of the pandemic on HIV testing globally. In a recent report from 44 high HIV burden countries across Africa, Latin America, the Caribbean, Asia and Europe, a reduction of HIV testing was observed across nearly all sites ranging from 26% to 44% [14]. While there was heterogeneity across sites, the same study observed an increase in test positivity ranging from 2% to 44%. Data from our group among the general Indian population demonstrated similar declines in tests performed and increases in test positivity [15]. From a single tertiary hospital in India, researchers found that HIV testing dropped 57% in 2020 compared to 2019 with a similar decline for new diagnoses [16]. An analysis of HIV services in sub‐Saharan Africa also saw HIV testing declines among general population clients; however, the decline associated with the initial COVID‐19 wave was modest (∼3%) compared to our findings with a faster rebound [17]. In contrast, COVID‐19 lockdowns in South Africa were found to correlate with nearly a 50% decline in testing in April 2020 with gradual improvement over time [18]. A qualitative study from Uganda demonstrated that access to HIV testing services was limited by travel restrictions, business closures and fear/stigma associated with visiting healthcare facilities during the pandemic [19]. Collectively, these data suggest potential prioritization of those most vulnerable to HIV or presenting with advanced disease. While there are more limited data from key populations, an online survey early in the pandemic among MSM across 20 countries found high levels of HIV testing interruptions [20]. Interestingly, our data suggest that test positivity was not higher during the initial waves of the pandemic (when testing declined); rather there was a long lag—a year—before the increase was evident. We cannot discern whether this delayed increase in positivity reflects changes in risk behaviour during the pandemic or delayed testing. Continued monitoring is needed to understand whether these trends are sustained.

We observed similar declines in the uptake of other preventive services during the first wave of the pandemic, including STI testing, counselling and condoms, though most rebounded to pre‐pandemic levels and did not decline as substantially during the second wave. Moreover, among PLHIV surveyed, 50% of MSM were unable to access STI testing and partner HIV testing services when needed. Unfortunately, we did not have reliable data to assess COVID‐19 impacts on MOUD utilization, but it was encouraging that among PLHIV surveyed, more than 90% of PWID who required MOUD reported being able to access this service. In India and across other contexts [21], MOUD programs adapted to accommodate COVID‐19 precautions (e.g. dispensation of 5–7 days’ worth of medication to take at home as opposed to daily observed therapy at a service provider) and survey data support the potential positive impacts of programmatic changes in response to the pandemic.

Unfortunately, we were unable to directly evaluate the pandemic's impact on ART refills—as most ICCs do not dispense ART—or viral suppression. Our service utilization data indicated modest declines in the number of HIV care referrals and linkage, likely representing a small fraction of HIV care received at government facilities. Qualitative data from other cities around India supported the success of MMD (vs. single month as was standard) as well as field/home delivery of ART [10], suggesting that the impacts on ART might have been less than preventive and testing services. Analyses from sub‐Saharan Africa found similar patterns, with a substantially smaller impact on ART use compared to HIV testing [17, 18]. Among MSM living with HIV across 20 countries, 20% reported they were unable to see their HIV provider because of pandemic mitigation strategies—notably lower than reported interruptions in HIV testing [20]. Data from our survey among PLHIV suggested that PWID faced higher barriers to accessing HIV treatment services than MSM. This is not surprising as PWID lag behind MSM with respect to the HIV care continuum in these same cities across India [22]. However, the majority of PLHIV did not report treatment interruption, consistent with what was expected based on national program changes.

There are several limitations to these data. First, we only have service data available from a single centre in each city. It is possible that as a result of the pandemic, individuals visited different facilities—those more accessible or in their hometown after mass migration induced by the nationwide lockdown in March 2020. However, pandemic closings/lockdowns can be expected to have similar impacts on other programs as in the ICCs. The pandemic limited our ability to track ART refills from government centres and MOUD. The survey was only administered to PLHIV who had enrolled in a cohort study who may differ in terms of HIV care/treatment engagement. Moreover, we did not reach everyone in that cohort; it is possible that the most vulnerable individuals were not reached and thus barriers could be more substantial than what we reported. The survey, administered in June–August 2020, does not cover impacts from the second COVID‐19 wave.

## 5 CONCLUSIONS

In summary, the COVID‐19 pandemic and associated lockdowns, particularly during the first wave of the pandemic, dramatically impacted service utilization among PWID and MSM in multiple cities across India. It was encouraging that many services rebounded—though slowly—to near pre‐pandemic levels and impacts were less severe during the second wave versus the first wave of the pandemic. Some changes implemented by the Indian government may have attenuated impacts and should be considered to optimize engagement beyond the pandemic. Of particular concern is the increase in new diagnoses during the second wave of the pandemic, which could signal increased HIV transmission and patients presenting with advanced disease among vulnerable groups.

## COMPETING INTERESTS

The authors declare that they have no competing interests.

## AUTHORS’ CONTRIBUTIONS

NPM–data analysis and interpretation; SA and JJB–data acquisition and interpretation; AKS, SSS, GML, DDC and SHM–study concept and design. All authors have read and approved the final manuscript.

## FUNDING

This research was supported by the National Institutes of Health: R01 DA041034, R01 MH89266, R01 DA032059 and K24 DA035684.

## Supporting information


**Figure S1** Percentage difference in any service utilization among unique integrated care centre (ICC) clients compared to January/February 2020

**Table S1** Any service use among unique MSM and PWID integrated care centre (ICC) clients over different time COVID‐19 pandemic periods in India, by population group
**Table S2** HIV testing among unique MSM and PWID ICC clients over different time COVID‐19 pandemic periods in India, by population group

## Data Availability

The authors confirm that all data underlying the findings are fully available upon request after a concept review from amcfall2@jhu.edu and smehta@jhu.edu.
