# Supplementary material for: Impact of the COVID‐19 pandemic on HIV prevention and care services among key populations across 15 cities in India: a longitudinal assessment of clinic‐based data
Source: J Int AIDS Soc. 2022 Jul 11;25(7):e25960. doi: 10.1002/jia2.25960 (PMC9273869; doi:10.1002/jia2.25960)

Supplementary Figure 1 Percentage difference in any service utilization among unique integrated care center (ICC) clients compared to January/February 2020

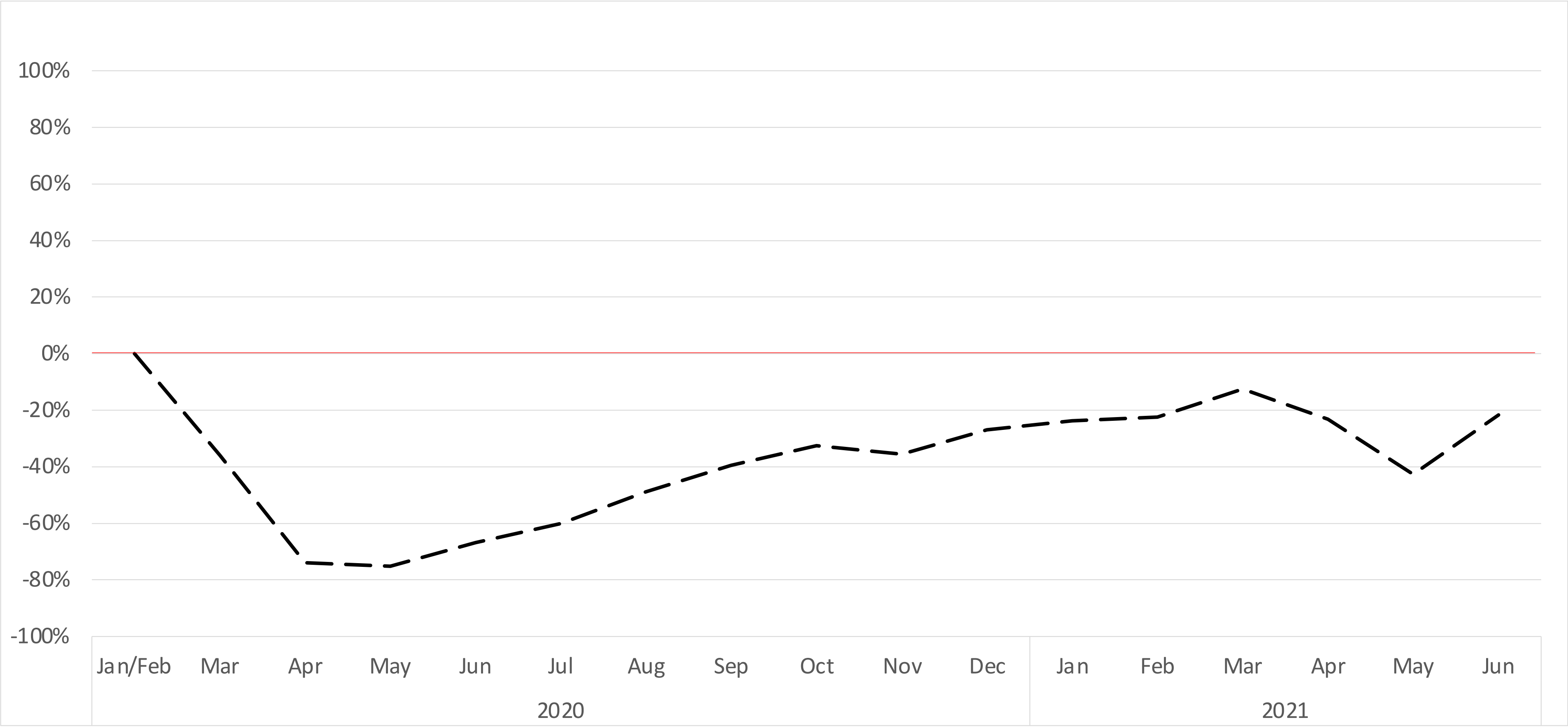

Supplement: Supplementary file 1 — Figure S1 Percentage difference in any service utilization among unique integrated care centre (ICC) clients compared to January/February 2020 [file JIA2-25-0-s002.pdf]
