# Supplementary material for: Impact of the COVID‐19 pandemic on HIV prevention and care services among key populations across 15 cities in India: a longitudinal assessment of clinic‐based data
Source: J Int AIDS Soc. 2022 Jul 11;25(7):e25960. doi: 10.1002/jia2.25960 (PMC9273869; doi:10.1002/jia2.25960)
Supplement: Supplementary file 2 — Table S1 Any service use among unique MSM and PWID integrated care centre (ICC) clients over different time COVID‐19 pandemic periods in India, by population group Table S2 HIV testing among unique MSM and PWID ICC clients over different time COVID‐19 pandemic periods in India, by population group [file JIA2-25-0-s001.docx]

**Methods for Supplementary Tables 1 and 2**

We used negative binomial regression models to statistically compare temporal trends of ICC service utilization during different COVID-19 pandemic periods in India. For the model outcome, counts of unique clients accessing services were summarized monthly by site. The explanatory variable of interest was time (months), starting at January/February 2020 and then each month after until June 2021 (i.e., March 2020, April 2020, May 2020… June 2021). Time was modeled linearly with 3 knots at: 1) May 2020, 2) March 2021, and 3) May 2021 to make 4 periods: 1) January-May 2020, 2) May 2020-March 2021, 3) March-May 2021, and 4) May-June 2021. These knots/time periods align with the COVID-19 pandemic waves and periods of mitigation strategies in India. The negative binomial regression model provided a within period rate ratio - the monthly change in service utilization in that period - as well a comparison (ratio) of the period rate ratio to the preceding period’s rate ratio. Standard errors were adjusted for within-site correlation.

**Supplementary Table 1** Any service use among unique MSM and PWID integrated care center (ICC) clients over different time COVID-19 pandemic periods in India, by population group

|  | **Within period rate ratio^1^** | **95% CI** | **Preceding period rate ratio comparison^2^** | **95% CI** |
| --- | --- | --- | --- | --- |
| **MSM** |  |  |  |  |
| Period 1: Jan-May 2020 | 0.69 | 0.61, 0.77 | -- | -- |
| Period 2: May 2020-March 2021 | 1.11 | 1.07, 1.15 | 1.61 | 1.40, 1.86 |
| Period 3: March-May 2021 | 0.63 | 0.53, 0.75 | 0.57 | 0.47, 0.68 |
| Period 4: May-June 2021 | 1.56 | 0.90, 2.71 | 2.49 | 1.22, 5.06 |
| **PWID** |  |  |  |  |
| Period 1: Jan-May 2020 | 0.69 | 0.63, 0.76 | -- | -- |
| Period 2: May 2020-March 2021 | 1.15 | 1.11, 1.18 | 1.66 | 1.48, 1.86 |
| Period 3: March-May 2021 | 0.79 | 0.73, 0.85 | 0.69 | 0.63, 0.75 |
| Period 4: May-June 2021 | 1.32 | 0.99, 1.77 | 1.69 | 1.19, 2.39 |

1: Within period monthly change in service utilization; 2: Period’s rate ratio compared to preceding rate ratio

MSM: men how have sex with men; PWID: people who inject drugs; ICC: integrated care centers; CI: confidence interval

**Supplementary Table 2** HIV testing among unique MSM and PWID ICC clients over different time COVID-19 pandemic periods in India, by population group

|  | **Within period rate ratio^1^** | **95% CI** | **Preceding period rate ratio comparison^2^** | **95% CI** |
| --- | --- | --- | --- | --- |
| **MSM** |  |  |  |  |
| Period 1: Jan-May 2020 | 0.59 | 0.46, 0.75 | -- | -- |
| Period 2: May 2020-March 2021 | 1.21 | 1.13, 1.31 | 2.05 | 1.50, 2.81 |
| Period 3: March-May 2021 | 0.40 | 0.27, 0.58 | 0.33 | 0.21, 0.51 |
| Period 4: May-June 2021 | 3.16 | 1.48, 6.77 | 7.93 | 2.63, 23.9 |
| **PWID** |  |  |  |  |
| Period 1: Jan-May 2020 | 0.77 | 0.66, 0.90 | -- | -- |
| Period 2: May 2020-March 2021 | 1.09 | 1.04, 1.14 | 1.41 | 1.16, 1.72 |
| Period 3: March-May 2021 | 0.76 | 0.66, 0.87 | 0.70 | 0.59, 0.83 |
| Period 4: May-June 2021 | 2.59 | 1.35, 4.98 | 3.42 | 1.61, 7.24 |

1: Within period monthly change in service utilization; 2: Period’s rate ratio compared to preceding rate ratio

MSM: men how have sex with men; PWID: people who inject drugs; ICC: integrated care centers; CI: confidence interval
